# The Role of Lipid Rafts in Cancer Cell Adhesion and Migration

**DOI:** 10.1155/2012/763283

**Published:** 2011-12-29

**Authors:** Toshiyuki Murai

**Affiliations:** Department of Microbiology and Immunology, Graduate School of Medicine, Osaka University, 2-2 Yamada-oka, Osaka, Suita 565-0871, Japan

## Abstract

Lipid rafts are cholesterol-enriched microdomains of the cell membrane and possess a highly dynamic nature. They have been involved in various cellular functions including the regulation of cell adhesion and membrane signaling through proteins within lipid rafts. The dynamic features of the cancer cell surface may modulate the malignant phenotype of cancer, including adhesion disorders and aggressive phenotypes of migration and invasion. Recently, it was demonstrated that lipid rafts play critical roles in cancer cell adhesion and migration. This article summarizes the important roles of lipid rafts in cancer cell adhesion and migration, with a focus on the current state of knowledge. This article will improve the understanding of cancer progression and lead to the development of novel targets for cancer therapy.

## 1. Introduction

The alternation of cell adhesion and highly migratory behavior are the most prominent features of cancer cells, and play critical roles in their aggressive invasion and metastatic spread [1]. These processes appear to be facilitated by remodeling of the extracellular matrix (ECM) of the tumor microenvironment and adhesion molecules at the cancer cell surface and affected by both the interaction between ECM and adhesion molecules and by growth factor signaling [2, 3]. The proteolytic ectodomain cleavage and release (shedding) of adhesion molecules are also critical regulatory steps in cancer cell adhesion and migration [4, 5].

 To date, cholesterol-enriched membrane microdomains called “lipid rafts” have been implicated in a variety of pathogeneses [6]; neurological diseases including Alzheimer's [7], Parkinson's [8], and prion diseases [9]; cardiovascular diseases; immune disorders such as systemic lupus erythematosus [10] and HIV infection [11]. Lipid rafts have been also implicated in signaling pathways in cancer progression [12], but how these microdomains affect the adhesion and migration of invasive cancer cells remains obscure. In this paper, recent findings on the roles of lipid rafts in cancer cell adhesion and migration will be reviewed.

## 2. Lipid Raft Structure

The prevailing model of cellular membrane structure was proposed by Singer and Nicolson, and this model is known as the fluid mosaic model, where globular proteins float in a lipid bilayer with a basic structure [13]. Later, the model was improved by Simons and van Meer, who suggested the existence of microdomains or “rafts” in the plasma membrane of epithelial cells [14]. In the current understanding of the lipid raft model, cholesterol- and sphingolipid-enriched microdomains of the plasma membrane exhibit a biophysical state comparable to the liquid-ordered phase floating in the liquid-disordered phase of the membrane [15]. One subtype of lipid rafts exists in flask-shaped plasma membrane invaginations called caveolae [16].

 Lipid rafts consist of assemblies of cholesterol, sphingolipids including sphingomyelin and gangliosides, and certain types of proteins [15]. Sphingolipids contain saturated fatty acyl chains in their structure, thereby allowing cholesterol to be tightly intercalated in the sphingolipid assemblies to form liquid-ordered microdomains. The most important properties of lipid rafts are that they are small, dynamic, and heterogeneous and can include or exclude proteins to variable extents [17, 18]. Proteins with raft affinity include glycosylphosphatidylinositol-anchored proteins, palmitoylated proteins, doubly acylated proteins, such as Src family kinases (SFKs), and transmembrane proteins such as CD44. Lipid rafts have been implicated in various physiological cellular processes, such as protein membrane trafficking and signal transduction [18, 19].

## 3. Tools for Lipid Raft Analyses

### 3.1. Lipid Raft Markers

Lipid rafts can be fractionated as detergent-resistant membrane (DRM) fractions using nonionic detergents such as Triton X-100 [18, 20]. Cholesterol- and sphingolipid-enriched rafts are insoluble in Triton X-100 at 4°C and float to a low-density area during gradient centrifugation. Notably, the constitution of DRM is affected by the type and concentration of detergents, and lipid rafts contained in DRM are nonnative aggregates. Marker molecules for lipid rafts are frequently used in biochemical and cytochemical analyses. The ganglioside GM1 is the most commonly used marker among putative lipid components of rafts; it is detected using the GM1-binding molecule, cholera toxin subunit B (CTxB) [21]. Protein markers such as caveolins and flotillins are also used for identifying lipid rafts [22].

### 3.2. Cholesterol Clathrate

Membrane cholesterol serves as a spacer for the hydrocarbon chains of sphingolipids and maintains the assembled microdomains of lipid rafts. Thus, cholesterol depletion leads to the disorganization of lipid raft structure. Methyl-*β*-cyclodextrin (M*β*CD), a torus-shaped cyclic oligosaccharide composed of 7 d-glucopyranosyl units linked by *α*-1,4 glycosidic bonds, is used to extract membrane cholesterol selectively and to disrupt lipid rafts [23]. M*β*CD is a practical tool for membrane studies as it neither binds to nor inserts into the plasma membrane. M*β*CD-mediated manipulation of membrane cholesterol is now a standard methodology in the research of lipid rafts [18, 24]. However, M*β*CD may deplete cholesterol from both the raft and nonraft domains of the membrane as well as alter the distribution of cholesterol between the plasma membrane and organelle membranes under high concentrations (i.e., >10 mM). Thus, it is recommended that a cholesterol-repletion experiment using cholesterol-M*β*CD complex and raft disruption with other cholesterol-sequestering agents as described below would be performed for confirmation.

### 3.3. Cholesterol-Binding Antibiotics

Filipin, a fluorescent polyene macrolide antibiotic from *Streptomyces filipinensis*, binds cholesterol and disperses it in the membrane. Filipin is thus used as a cholesterol probe and a cholesterol sequestration agent in the research of lipid rafts [25, 26]. Other than filipin, nystatin and amphotericin are also used in lipid-raft analyses.

### 3.4. Inhibitors for Cholesterol Biosynthesis

Statins are widely used inhibitors of 3-hydroxy-3-methylglutaryl-CoA reductase, the key rate-limiting enzyme in the biosynthesis of cholesterol. Statins lower cellular cholesterol content and thus are useful in the analysis of lipid-raft function. Prevention studies using statins have confirmed its significance in the prevention of cardiovascular diseases [27]. It has also been demonstrated that statins may be an effective preventive medicine for neurodegenerative diseases, including Alzheimer's disease [28]. Although the various population-based reports of the effects of statins on cancer are controversial, recent epidemiologic studies suggest that statins inhibit the progression of certain cancers [29]. Recent evidence suggests that statins blocked the adhesion and migration processes of cancer cells [30, 31]. Cholesterol reduction is a potential therapy for suppressing cancer cell adhesion and migration.

## 4. Lipid Rafts and Proteolytic Processing of Adhesion Receptors

CD44 is a major cell adhesion molecule expressed in cancer cells and implicated in cancer cell adhesion, migration, and metastasis [32–34]. A number of reports have demonstrated that CD44 is present in lipid rafts [35–40], but the role of lipid rafts in cancer cell adhesion and migration has not been elucidated.

**Figure 1:**
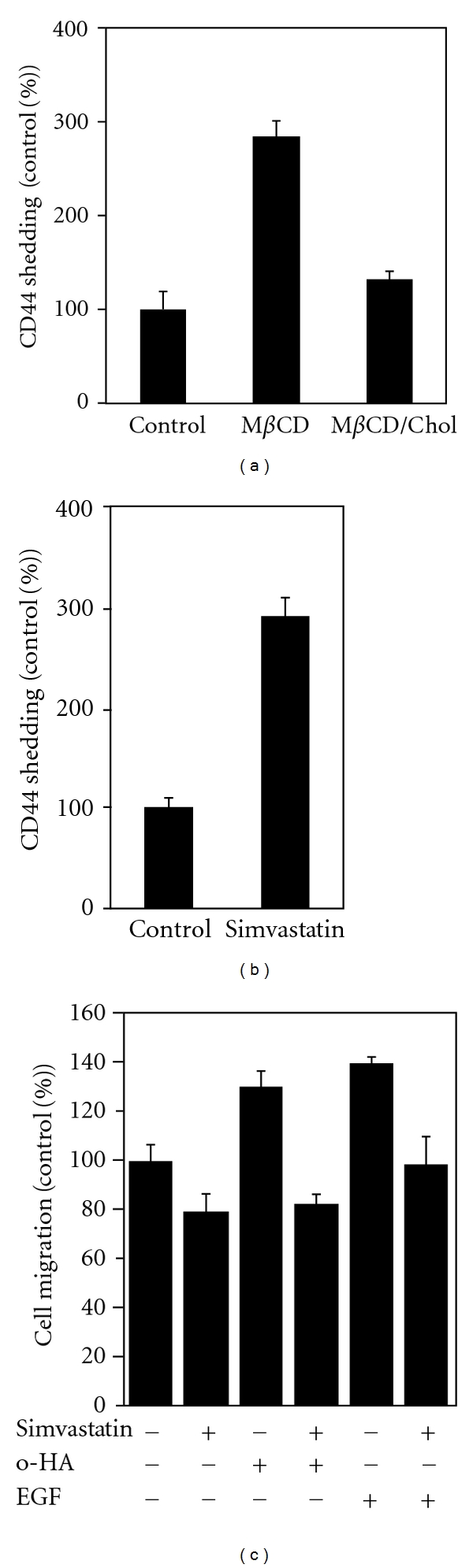
Cholesterol lowering stimulates CD44 shedding and suppresses cancer cell migration. (a) Modulation of cellular cholesterol affects CD44 shedding from human glioma cells. Cells were cholesterol-depleted (M*β*CD), cholesterol-replenished (M*β*CD/Chol), or left untreated (control), and CD44 shedding was assessed by measurement of soluble CD44 in the culture medium. (b) Effect of simvastatin on CD44 shedding. Cells were incubated in the presence or absence of simvastatin, and CD44 shedding was assessed by measurement of soluble CD44 in the culture medium. (c) Effect of simvastatin on CD44-dependent cell migration. Cells were incubated in the presence or absence of simvastatin, and treated with hyaluronan oligosaccharides (o-HA) or EGF.

**Figure 2:**
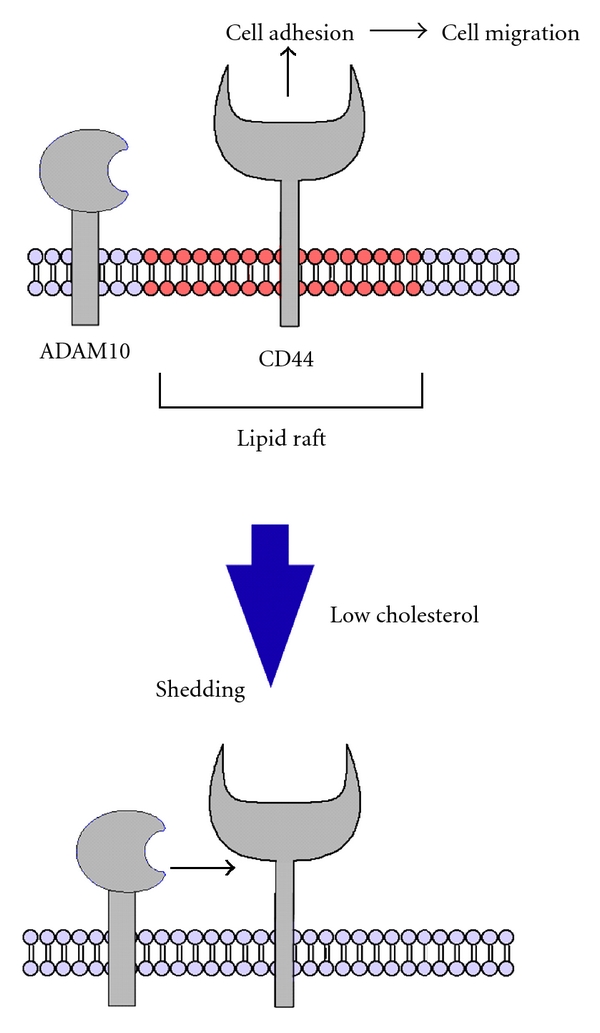
A putative model of the lipid-raft-related cancer cell adhesion and migration.

 Recently, it was demonstrated that lipid rafts play a crucial role in the localization and functionality of CD44, which regulates cancer cell adhesion and migration [31]. Treatment of human glioma cells with the lipid-raft-disrupting agent M*β*CD resulted in an increase in CD44 shedding (Figure 1(a)) [31]. Similar patterns are observed when cells were treated with another lipid-raft-disrupting agent, filipin, and also in the case of pancreatic cancer cells. Analyses of Triton X-100 solubility of CD44 and its processing enzyme, a disintegrin and metalloproteinase 10 (ADAM10), revealed that CD44 was present in both Triton-X-100-insoluble and Triton-X-100-soluble fractions of untreated cells, whereas ADAM10 was largely in Triton-X-100-soluble fraction [30, 31]. Treatment with M*β*CD or filipin, however, led to loss of CD44 from the Triton-X-100-insoluble fraction. These results suggest that the perturbation of the ordered distribution of CD44 and ADAM10 on the membrane increased the probability of enzyme-to-substrate contact that leads to enhanced CD44 shedding. Membrane microdomains such as lipid rafts serve as platforms for the nanoscale assembly of membrane proteins. Simvastatin, one of the statins most frequently used in the clinical treatment of hypercholesterolemia, also enhanced CD44 shedding (Figure 1(b)). Moreover, simvastatin blocked the stimulation of glioma cell migration by hyaluronan oligosaccharides or epidermal growth factor (EGF) (Figure 1(c)) [41–43]. Taken together, these results suggest that lowering cholesterol levels may disturb the regulated CD44 membrane localization that is necessary for enhanced cancer cell adhesion and migration (Figure 2).

Recent studies on the shedding of various membrane proteins revealed that cholesterol depletion triggers the shedding of these molecules, including amyloid precursor protein (APP) [44], IL-6 receptor [45], CD30 [46], L1-CAM [47], and collagen types XVII [48] and XXIII [49]. It is especially noteworthy that APP and CD30 were found to be strongly associated with lipid rafts, whereas their processing enzymes, ADAM10 and ADAM17, respectively, are excluded from lipid rafts [44, 46]. These findings suggest that lipid rafts may play a critical role in regulating the accessibility of processing enzymes to their substrate proteins during both constitutive and regulated shedding [50].

 Na^+^-H^+^ exchanger interacts with CD44 in lipid rafts and may regulate cancer cell migration [39]. Complement component receptor gC1qR is a lipid raft protein that is concentrated in the lamellipodia along with CD44, regulating A549 lung adenocarcinoma cell migration and metastasis [51].

## 5. Cell Adhesion Signaling in Lipid Rafts

Integrins are transmembrane adhesion receptors composed of *α* and *β* subunits that facilitate the anchorage of cells to components of the ECM or bind to ligands on other cells to support cell-cell adhesion. Recent evidence suggests that the microorganization of lipids in the plasma membrane can affect integrin-mediated cellular functions [52]. Integrin-mediated cell adhesion to the ECM is regarded as one of the primary stages of SFKs' function. SFKs are activated in lipid rafts, and lipid-raft-specific inhibition of SFKs abrogates adhesion of breast cancer cells [53]. The transmembrane phosphoprotein, Cbp, a C-terminal Src kinase-binding protein, serves as a sensor of SFK activity in integrin-mediated cell adhesion signaling [54].

 CD44 is an important marker for various cancer stem cells (CSCs), such as pancreatic [55], breast [56], ovarian [57], colon [58], and bladder CSCs [59]. However, why CD44 is a CSC marker remains largely unknown. Recently, it was reported that lipid-raft-associated CD44 is required for the survival of CSCs in the suspension condition through CD44-SFK-integrin signaling, leading to tumor metastasis [60].

 Lipid rafts are necessary platforms for membrane receptor redistribution and the acquisition of a polarized phenotype during MCF-7 mammary adenocarcinoma cell migration [61]. Disruption of lipid rafts with M*β*CD abolishes lamellipodia formation and inhibits the chemotactic migration of MCF-7 cells [61].

## 6. Invasion Machinery and Lipid Rafts

A variety of invasive cancer cells form invadopodia, subcellular structures with ventral membrane protrusions that induce ECM degradation, a pivotal process in cancer invasion [62]. The ECM degradation activity of invadopodia is mainly mediated by membrane type 1-matrix metalloproteinase (MT1-MMP) concentrated at the surface of invadopodia [4]. Localization to lipid rafts is essential for the internalization of MT1-MMP. Lipid rafts are required for invadopodia formation in breast cancer cells and ECM degradation [63]. Caveolin-1 is predominantly expressed in invasive breast cancer cell lines and is well correlated with invadopodia activity, implying that caveolin-1 plays important roles in the trafficking of the components of invadopodia including MT1-MMP [63].

##  Concluding Remarks

I have summarized here the nature of lipid rafts and their role in cancer cell adhesion and migration focusing on the current state of knowledge, although many questions about the nature of lipid rafts remain unsolved. Future studies may corroborate a variety of aspects of the role of lipid rafts in the regulation of adhesive and migratory properties of invasive cancer cells.

 The elucidation of the mechanism underlying the lipid-raft-mediated regulation of cancer cell adhesion and migration will provide new insights into the mechanism of cancer invasion and metastasis and also provide a wealth of new targets for cancer prevention and therapy for clinical medicine.

## Abbreviations

ADAM: A disintegrin and metalloproteinase
APP: Amyloid precursor protein
CSC: Cancer stem cells
CTxB: Cholera toxin subunit B
DRM: Detergent-resistant membrane
ECM: Extracellular matrix
M*β*CD: Methyl-*β*-cyclodextrin
SFK: Src family kinase
EGF: Epidermal growth factor
MT1-MMP: Membrane type 1-matrix metalloproteinase.
